# Effect of Particle Size Ratios on the Physical and Chemical Properties of Surgical‐Grade Calcium Sulfate Hemihydrate

**DOI:** 10.1111/os.12569

**Published:** 2019-12-21

**Authors:** Pan Hu, Deng‐xing Lun, Peng‐sheng Wang, Zhi‐ming Tu

**Affiliations:** ^1^ Department of Orthopedics The Third Hospital of Hebei Medical University Shijiazhuang Hebei China; ^2^ Department of Spine Surgery Weifang People's Hospital Weifang Shandong China; ^3^ Beijing Ceramics Biotechnology Co. Ltd Beijing China; ^4^ Department of Spine Surgery The Second Xiangya Hospital of Central South University Changsha China

**Keywords:** Calcium sulfate dihydrate, Calcium sulfate hemihydrate (CSH), Compressive strength, Setting time

## Abstract

**Objectives:**

To analyze the optimum particle size or formula ratio of surgical‐grade calcium sulfate (CS) for appropriate compressive strength, setting time, and vitro degradation rate.

**Methods:**

Three types of calcium sulfate hemihydrate (CSH) particles with diameters of 0–37.5 μm, 37.5–75 μm, and >75 μm were screened. Based on formulation ratio of different particles, this topic is divided into 10 groups by the unconstrained third‐order simplex lattice mixing design scheme in formula design experiment. The optimum formulation ratio of particle diameter for compressive strength, solidification time, and degradation rate *in vitro* was analyzed.

**Results:**

When the percentage of the particle diameter of CS with 0–37.5 μm, 37.5–75 μm and >75 μm are 55.0%, 17.4%, and 27.6% respectively, the compressive strength of the test sample is the highest, which is 14.16 MPa. When the percentage of the particle diameter of CS with 0–37.5 μm, 37.5–75 μm, and >75 μm are 0.00%, 0.00%, and 100.00% respectively, the initial setting time of the sample is the longest, which is 410.0 s. When the percentage of the particle diameter of CS with 0–37.5 μm, 37.5–75 μm, and >75 μm are 0.00%, 0.00%, and 100.00% respectively, the final setting time of the sample is the largest, and the final setting time of the sample is 460.00 s. When the percentage of the particle diameter of CS with 0–37.5 μm, 37.5–75 μm, and >75 μm are 0.00%, 0.00%, and 100.00% respectively, the degradation rate of the sample *in vitro* is the slowest, which is 18.8%.

**Conclusion:**

The morphological structure of surgical‐grade CS can affect compressive strength, setting time, and in vitro degradation rate. Surgical CS should be prepared based on different uses.

## Introduction

The ideal bone graft material should possess good osteogenesis, osteoinductivity, and osteoconductivity^1^, ^2^, ^3^. The most commonly‐used bone graft materials for now include autograft, allograft, and artificial bone graft^2^. The autograft is the gold standard of the reconstruction surgery, and it has those three crucial advantages mentioned above. However, the related complications of the donated part might be difficult to avoid and the sources of autograft are limited^3^, ^4^. Speaking of the allograft, its resource is abundant, and it has the microstructure of natural bone, which could facilitate the ingrowth of osteoblasts. But the quality of the donors is hard to control, and there are still risks of disease propagation. The artificial bone graft is used to simulate the bone‐growing progress. The artificial bone is absorbed by the surrounding original bone tissues. By the general growing process, it fuses with the surrounding bone and finally forms a complete part of bone without original defects. Although there is a wide range of sources of the artificial bone, it requires the stringent demand of manufacturing processes. At present, the materials used as artificial bone include calcium sulfate (CS), calcium phosphate (TCP or HA), biological glass and their composites.

CS has a good biocompatibility^5^, ^6^, ^7^, ^8^, ^9^. It can be completely absorbed by the human body without leaving any residual tissues, hence it does not cause inflammatory responses^5^. However, early studies found that the resorption rate of CS is obviously higher than that of the bone growth, which made it limited in clinical applications. Thus, it could only be applied as a drug carrier for injected patients^10^. With long‐term technological development, CS can be applied not only for bone repairment, but also as a plasticizer. It can be mixed with autograft, allograft, and bio‐ceramic materials as bone cement, and can be randomly molded according to the defect shape in operations. In addition, it can also be injected at the bone defect part, and act as a drug carrier for the delivery of anti‐neoplastic or anti‐tuberculosis drugs. It can carry various types of antibiotics to deal with open fracture and infectious bone defects. It is presently the only bio‐ceramic material that combines two or more properties for medical use.

At present, surgical grade calcium sulfate mainly depends on import and it is expensive. It is still in the beginning line for Chinese researchers to learn how to fabricate the CS products like Osteoset from Wright, US, or Stimular from Biocomposites, UK. In addition, there is a lack of effective and systematic studies to evaluating there products. To our knowledge, recent researches are mainly focused on how to select the surgical‐grade CSH material with good biocompatibility, degradation rate, and set timing.

The morphological structure of surgical‐grade calcium sulfate hemihydrate (CSH) can affect its biological performance together with its physical and chemical properties^11^. Especially, the various particle sizes can obviously alter its compressive strength and *in vitro* degradation rate. It is normally thought that the smaller particle size would induce larger surface‐volume ratio. When the substance is set and hardened, a large amount of free water rapidly turns into crystal water, resulting in decreasing amount of free water between molecules and high compressive strength. However, a smaller particle size would further lead to rapid *in vivo* resorption rate. Thus, it is one of the most essential and difficult subjects to discover and select the particle with proper size, in order to satisfy the mechanic requirement and achieve the viable degradation rate.

Recently, there have been a few studies about the effect of particle size of CSH on its physical and chemical properties, however, the experimental designs were largely limited. Most studies grouped the particles with single sizes and did not distribute different sizes of particles with the certain ratio into one group^12^, ^13^. Moreover, despite the studies of different particle sizes, most experimental results were affected subjectively by the designer. The conclusions were obtained mainly by subjective observations, while objective statistical methods for accurate results were rarely applied.

Thus, the purpose of this study was to objectively observe the effect of CSH mixtures with different particle sizes on (i) its compressive strength, (ii) setting time, and (iii) *in vitro* degradation performance.

## Materials and Methods

### *Instruments and Materials*

Experimental materials: purified water (self‐produced), phosphate buffered solution (PBS) with the pH value of 7.4 ± 0.1.

Experimental instruments: Incubator (MJ‐70‐I mold incubator, Shanghai Qixin Scientific Instrument Ltd.); Computerized universal test machine (WDW‐10, Jinan Chuanbai Instrument & Equipment Ltd., maximum range 10 kN); Air‐dry oven (GZX‐9030MBE, Medical Equipment Factory of Shanghai Boxunshiye Ltd., maximum range 250 °C); Analysis balance (JA2013A, Beijing Astronautic Metrology Research Institute, maximum range 200 g); and New‐standard Vicat apparatus (WKY, Shanghai Luda Experimental Instrument Ltd.).

### *Experimental Design and Grouping*

The experiment is designed according to the **“non‐constraint three‐order simplex‐lattice formula”** from the formula‐designing experiment. The CSH samples are distributed into 10 groups with various ratios of particle sizes, shown in Table 1.

**Table 1:** The grouping method according to the “non‐constraint three‐order simplex‐lattice formula” from the formula‐designing experiment

Groups	X_1_ *	X_2_	X_3_
1	1	0	0
2	0	1	0
3	0	0	1
4	1/3	2/3	0
5	1/3	0	2/3
6	2/3	1/3	0
7	2/3	0	1/3
8	0	1/3	2/3
9	0	2/3	1/3
10	1/3	1/3	1/3

* X_1_, X_2_, and X_3_ represent the weight percentage of CSH particle at the diameters of 37.5 μm, 37.5–75 μm and 75–150 μm respectively. 1/3 and 2/3 represent the ratios of CSH at this particle size within the entire sample.

**Figure 1:**
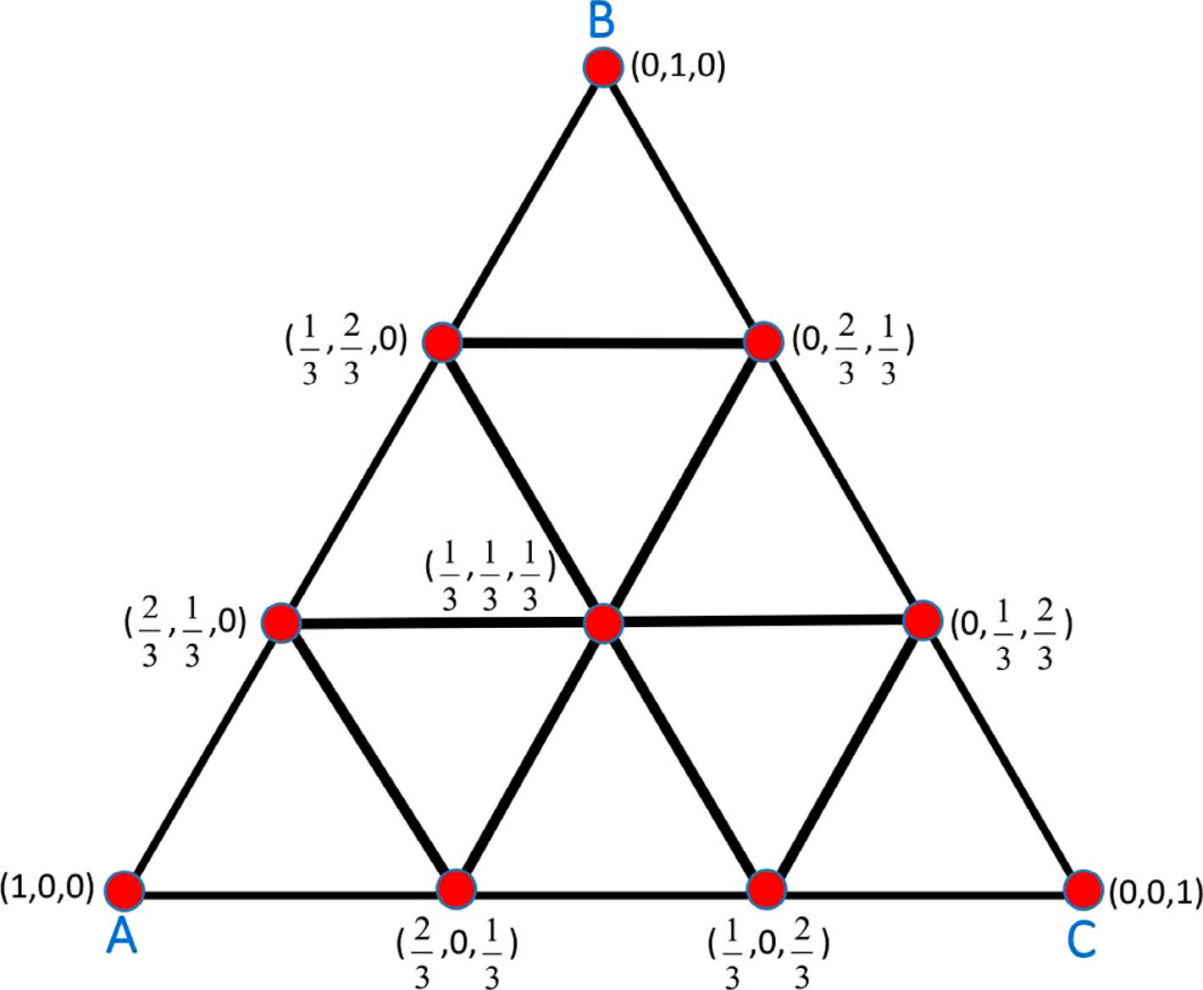
The experimental design of non‐constraint three‐order simplex‐lattice formula. The three sides of an equilateral triangle (with the height at one unit) is trisected to generate a three‐order lattice point set, recorded as a {3, 3} simplex‐lattice design, in which the former three represents the number of standard simplex vertex m, and the latter indicates the trisection number of each side d. This design includes 10 groups of experiments.

The experimental design method is shown in Fig. 1. In this experiment, the restrictive conditions of the percentage compositions for A(X_1_), B(X_2_), and C(X_3_) gives:

$$0\leq X_{1}\leq 1,0\leq X_{2}\leq 1,0\leq X_{3}\leq 1,\text{and}\;X_{1}+X_{2}+X_{3}=1.$$

As for the parameters of the compressive strength, initial setting time, final setting time and *in vitro* degradation tests, the basic form of quasi‐solving regression equation gives:

$$Y=a_{0}+a_{1}•X_{1}+a_{2}•X_{2}+a_{3}•X_{1}•X_{2}+a_{4}•X_{1}^{2}+a_{5}•X_{2}^{2}+a_{6}•X_{1}•X_{2}^{2}+a_{7}•X_{1}^{2}•X_{2}+a_{8}•X_{1}^{3}+a_{9}•X_{2}^{3}$$

### *Material Preparation*

1. **Particle screening.** The sieves with 100, 200, and 400 meshes are applied to screen the particles. The particles range from 0 to 37.5 μm, 37.5 to 75 μm, and 75 to 150 μm, divided by the electric sieve machine.
2. **Grouping.** Adjust the components in each group according to Table 1.
3. **Solid–liquid ratio.** The ratio between solid and liquid components is 1:0.7. The components are stirred evenly to obtain the injectability.

### *Biomechanical Testing Method*

1. **Preparation of the standard sample.** The diameter and height of the cylindric sample is respectively 10.0 ± 0.1 mm and 15.0 ± 0.1 mm. The parallelism between the upper and lower plane of the sample should be no more than 0.1 mm. The perpendicularity from the lateral plane to the upper and lower plane should be no more than 0.1 mm.
2. **Sample drying.** The sample is placed in the thermostat for 24 h at 37°C to dry completely.
3. **Sample setting and loading.** The cylindrical pressure vessel of the sample should have the hardness of no less that 300 HV (≥ 30 HRC). Its thickness should be no less that 10 mm, and the surface area should be over four times larger than the sample. The toughness (Ra) of the cylindrical pressure vessel and the section of the sample should be no more than 0.40 μm, and the parallelism should be no more than 0.01 μm. The sample should be placed at the center of the substrate, so that the substrate, the sample, the sphere, and the cylindrical pressure vessel should be aligned vertically along the loading line. The loading speed is kept at 0.5 ± 0.05 mm/min.
4. **Compressive strength testing.** During the compressive strength test, the loading displacement curve should be plot from the beginning of the test to fracture. The maximum load and maximum displacement are respectively recorded for each loading test, in which the former is used to calculate the compressive strength. The equation gives that:

$$\sigma =\frac{P_{\max}}{S},$$

in which σ (Pa) is the compressive strength, P_*max*_ (N) is the maximum load, and S (m^2^) is the load‐bearing area per unit.

**5. Recording and calculating.** The maximum load of e samples in each group are recorded. The maximum surface area and compressive strength of each sample are calculated according to the equation above, and the average values are obtained. The same method is applied to calculate the values of other groups.

### *Testing Method of the Setting Time*

1. **Preparation of the standard sample.** The mixture is prepared in order, according the standard ratio. The mixture is then poured into a cylindrical mold with the diameter of 2 cm and the height of 4 cm. Both ends are grinded smoothly. The apparatus with the mixture is placed into the humidity curing box, and the water introducing time is set as the beginning of the setting time. It should be ensured that the bone cement is homogeneously filled inside the mold without bubbles. Each group of mixtures are divided and fabricated into three samples.
2. **0‐point adjustment.** The needle of the Vicat apparatus for initial and final setting time are respectively adjusted so that it could target the 0‐point when contacting the baseboard.
3. **Initial setting time testing.** The sample is placed in the humidity curing box for 2 min and tested the first time. When testing, the testing mold is taken out from the humidity curing box. The needle is lowered to contact the sample surface. After screw is fastened for 1–2 s, the needle is dropped to be sunk free inside the sample. The decreasing value of the needle is then observed and recorded. When it approaches the initial setting time, the value of the decreasing needle is recorded once per 0.5 min. The initial setting state of the sample is defined as the needle sinks inside the sample with the distance of the baseboard of 4 ± 1 mm. The initial setting time is recorded as the time period between the beginning of the adjustment and the initial setting state, accurate to 30 s.
4. **Final setting time testing.** In order to accurately observe the state of the needle sinking into the mold, a circle component is set on the final setting needle. After the initial setting time is observed, the mold and slurry are immediately taken out parallelly from the glass board, and turned over oppositely onto the board. When it approaches the final setting time, the value of the decreasing needle is recorded once per minute. It is defined as the final setting state when the needle is sunk inside the sample at 0.5 mm, i.e. a mark cannot be left in sample. The final setting time is recorded as the time period between beginning of adjustment and the final setting state, accurate to 30 s.
5. **Measuring and recording.** The initial and final setting time is recorded and calculated in average.

### *Measuring Method of* In vitro *Degradation Test*

1. **Preparing the standard sample.** The mixtures of each group are manufactured into three semi‐spherical samples with the dimeter of 6 mm, and dried at 37°C for 24 h.
2. **Degradation testing *in vitro*.** For each group, three sterile bottles are used to add, respectively, 10 mL PBS inside. Then three samples are placed inside the bottles. It should be ensured that the samples are completely immersed in the PBS. The bottles are then sealed and placed inside the thermostat at 37°C. The samples are taken in 5, 10, 15, 20, 25, 30, 35, and 40 days respectively. After placed inside the drying oven, the weights of samples in each group are measured and taken in average. Then the samples are further placed inside the weighing bottles with newly‐added 10 mL PBS and placed again in the thermostat.
3. **Recording and calculating.** The weights at 10 monitoring time points are recorded respectively as *M*_*0*_, *M*_*5*_, … *M*_*40*_. The equation of degradation rate *R*_*i*_ gives:

$$R_{i}=\frac{M_{0}-M_{i}}{M_{0}},$$

where *M*_*0*_ is the original weight, and *M*_*i*_ is the weight at *i* days.

### *Statistical Methods*

The data are analyzed by SPSS (version 23.0), in which all the data are in the form of “average ± standard deviation”. For the comparison of compressive strength, initial setting time, final setting time, and *in vitro* degradation rate respectively, the one‐way ANOVA method is adopted. Between‐groups comparison applies LSD *post hoc* tests, and the statistical significance is defined as *P* < 0.05.

## Results

### *Biomechanical Test Results*

#### *Compressive Strength Results*

As seen in Table 2, the compressive strength of single diameter‐value particles is maximum at the diameter of 0–37.5 μm, with the strength of 12 MPa, while that of 75–150 μm is the minimum at 6.6 MPa. The compressive strength of particles at 37.5–75 μm is 9.2 MPa. This indicates that the larger the particle size, the weaker the compressive strength.

**Table 2:** The data results of compressive strength, initial setting time, final setting time and degradation rate of 10 groups of samples from the formula‐designing experiments

Groups	Content			Compressive strength (MPa)	Initial setting time (s)	Final setting time (s)	Degradation rate (%)
	X_1_	X_2_	X_3_				
1	1	0	0	12	**270** *	**320**	21.22
2	0	1	0	9.2	315	380	22.90
3	0	0	1	**6.6**	**410**	**460**	**18.81**
4	1/3	2/3	0	10.3	320	370	29.76
5	2/3	1/3	0	11.3	290	330	30.80
6	1/3	0	2/3	10.5	350	405	30.49
7	2/3	0	1/3	**13.3**	290	340	27.78
8	0	1/3	2/3	7.8	360	400	32.62
9	0	2/3	1/3	7.2	350	390	31.26
10	1/3	1/3	1/3	13.3	330	380	**32.66**

* The black‐styled data represents the maximum and minimum value of each aspect.

#### *Regression Equation, Regression Coefficients, and Optimal Design of the Compressive Strength*

The regression equation of compressive strength is derived as:

$$Y=6.60+10.35•X_{1}+10.70•X_{2}+61.20•X_{1}•X_{2}+8.55•X_{1}^{2}-27.9•X_{2}^{2}-54.00•X_{1}•X_{2}^{2}-86.40•X_{1}^{2}•X_{2}-13.5•X_{1}^{3}+19.8•X_{2}^{3}$$

This is obtained by the data from Table 2 introduced into the secondary tertiary equation, and the 10 regression numbers are calculated.

The optimum ratio was obtained by the “planning for solution” tool from Excel, which results that X_1_ = 0.550, X_2_ = 0.174, and X_2_ = 0.276. It means that the compressive strength is at its maximum of 14.16 MPa when the ratio of three types of particles sized are at the percentage of 55.0%, 17.4%, and 27.6%, respectively. However, when X_1_ = 0.000, X_2_ = 0.000, and X_3_ = 1.000, the compressive strength value is the smallest, at 6.60 MPa.

### *Initial Setting Time*

#### *Result Analyses of the Initial Setting Time*

Table 2 presents the results of the initial setting time of 10 groups of formula designing experiments. As for that of single‐sized particle groups, the shortest initial setting time is obtained at 270 s in the group of diameters with 0–37.5 μm. The diameter group of 75–150 μm results in the longest time at 315 s. Those data indicate that the increase of particle size could prolong the initial setting time.

#### *Regression Equation, Regression Coefficients, and Optimal Design of the Initial Setting Time*

The related data in Table 2 are brought into the secondary three‐times equation to establish 10 equations (seen in Appendix 2) and solve those 10 regression coefficients. The regression equation of initial setting time (Y) with the composition of each component gives:

$$Y=410.0-140.0•X_{1}-275.0•X_{2}+450.0•X_{1}•X_{2}-180.0•X_{1}^{2}+472.5•X_{2}^{2}-405.0•X_{1}•X_{2}^{2}-135.0•X_{1}^{2}•X_{2}+180.0•X_{1}^{3}-292.5•X_{2}^{3}$$

The optimum ratio was obtained by the “planning for solution” tool from Excel. It turned out that the initial setting time Y reaches the maximum of 410.0 s when X_1_ = 0.000, X_2_ = 0.000, and X_3_ = 1.000. It reaches the minimum of 268.9 s when X_1_ = 0.942, X_2_ = 0.000, and X_3_ = 0.058.

### *Final Setting Time*

#### *Result Analyses of the Final Setting Time*

Table 2 presents the results of the final setting time of 10 groups of formula designing experiments. As for that of single‐sized particle groups, the shortest final setting time is obtained at 320 s in the group of diameters with 0–37.5 μm. The diameter group of 75–150 μm results in the longest time at 380 s. Those data indicate that the increase of particle size could prolong the final setting time.

#### *Regression Equation, Regression Coefficients, and Optimal Design of the Final Setting Time*

The related data in Table 2 are brought into the secondary three‐times equation to establish 10 equations (seen in Appendix 3) and solve those 10 regression coefficients. The regression equation of final setting time (Y) with the composition of each component gives:

$$Y=460.0-95.0•X_{1}-305.0•X_{2}+562.5•X_{1}•X_{2}-292.5•X_{1}^{2}+450.0•X_{2}^{2}-472.5•X_{1}•X_{2}^{2}-270.0•X_{1}^{2}•X_{2}+247.5•X_{1}^{3}-225.0•X_{2}^{3}$$

The optimum ratio was obtained by the “planning for solution” tool from Excel. It turned out that the final setting time Y reaches the maximum of 460.0 s when X_1_ = 0.000, X_2_ = 0.000, and X_3_ = 1.000. It reaches the minimum of 316.28 s when X_1_ = 0.897, X_2_ = 0.103, and X_3_ = 0.000.

### *Results of In Vitro Degradation Rate Measurement*

#### *Primary Result Analysis of* In vitro *Degradation Rate*

The *in vitro* degradation rates of 10 groups of CSH samples at six various time points are shown in Table 3. Those samples with mixed sizes of particles could degrade thoroughly in 30 days, while the single‐sized samples still remained a few undissolved particles. In the 10 days from the beginning, the sample with the particle diameters of 75–150 μm had a slowest degradation rate of 18.81%. Other two single‐sized samples (which contain the diameters of 0–37.5 μm and 37.5–75 μm, respectively) had the similar degradation rate of 21.22% and 22.90%, respectively.

**Table 3:** *In vitro* degradation rates of 10 groups of CSH samples at various time points

Groups	V5 (%)*	V10 (%)	V15 (%)	V20 (%)	V25 (%)	V30 (%)	V35 (%)	V40 (%)
1	8.21	21.22	34.50	59.82	71.98	84.30	93.41	98.93
2	10.56	22.90	34.93	59.55	70.58	82.80	93.42	98.50
3	6.19	18.81	31.03	54.27	66.04	77.50	88.79	96.70
4	13.53	29.76	44.07	73.11	86.11	97.10	100	100
5	14.42	30.80	46.77	79.07	94.70	100	100	100
6	15.04	30.49	44.97	74.51	87.45	99.80	100	100
7	9.32	27.78	46.20	80.40	93.98	100	100	100
8	15.17	32.62	49.07	80.31	93.77	100	100	100
9	13.37	31.26	47.80	80.04	93.59	100	100	100
10	15.68	32.66	49.10	80.28	94.38	100	100	100

* Represents the degradation rate in fifth days.

#### *Regression Equation, Regression Coefficients, and Optimal Design of the* In Vitro *Degradation Rate*

The related data in Table 2 are brought into the secondary three‐times equation to establish 10 equations (seen in Appendix 4) and solve those 10 regression coefficients. The regression equation of degradation rate (Y) with the composition of each component gives:

$$Y=0.188+1.706•X_{1}+1.758•X_{2}-5.450•X_{1}•X_{2}-2.815•X_{1}^{2}-2.743•X_{2}^{2}+3.903•X_{1}•X_{2}^{2}+4.266•X_{1}^{2}•X_{2}+1.321•X_{1}^{3}+1.214•X_{2}^{3}$$

The optimum ratio was obtained by the “planning for solution” tool from Excel. It turned out that the degradation rate reaches the minimum of 18.8% when X_1_ = 0.000, X_2_ = 0.000, and X_3_ = 1.000. It reaches the maximum of 50.7% when X_1_ = 0.577, X_2_ = 0.433, and X_3_ = 0.000.

## Discussion

This study is the first time that the particle size ratios of CSH samples have been examined on its compressive strength, initial setting time, final setting time, and *in vitro* degradation rate, in order to meet the requirement of standards on the surgical‐grade CS proposed by American Society of Tests and Materials (ASTM)^14^. The study found that a smaller size CS particle would induce shorter initial and final setting time. This is possibly because a small particle size results in larger surface area and water‐containing area, and shortens the time of the hydration process. On the contrary, a larger particle size of CS sample would decrease the surface area and water‐contacting area, and further extend the hydration time. As for the compressive strength, the smaller particle size could induce larger surface area, and also a larger amount of water. When the solid–liquid ratio of the product is determined, this structure is likely to have smaller voids between particles, which enables it to form more crystal water with less free water left. After the drying process, a small void volume inside the bulk material would induce high compressive strength. The mixing application of small and large particles could enable the small particles to fill inside the voids of large ones inside the bulk sample, and further decrease the void volume after the drying process. This makes a higher compressive strength of the sample.

### *Relationship between Particle Size and Compressive Strength*

Zhang *et al*.^15^ found that the compressive strength of CSH is related to the aspect ratio and size of the CSH particles. When the aspect ratio or the particle size of CSH is smaller, the specific surface area of the particle gets higher. More water is transformed into crystal water inside the structure of hydrated calcium sulfate than a large particle‐ size CS sample. This induces a small volume of voids inside the material and a better compressive strength. Guan *et al*.^11^ discussed the relationship between the particle size distribution of CSH and the compressive strength of CSD. When the CSH particle sizes distributed in the range of 0–20 μm, 20–50 μm, and 90–140 μm, the compressive strength of obtained CSD was enhanced. The particle sizes of 50–90 μm and >140 μm would decrease the compressive strength. Thus, this study suggested that the CSH particles with the sizes of 0–20 μm, 20–50 μm, and 90–140 μm are crucial for the compressive strength, in which 20–50 μm and 90–140 μm particles have more positive effects than those with the size of 0–20 μm. The negative effect of particles with 50–90 μm diameters are stronger than those of >140 μm. Fu *et al*. ^12^ found that the maximum compressive strength was obtained at 17.2 MPa, when CSH samples with small particle sizes were mixed and hardened with water at a liquid/powder ratio of 0.6. The middle‐size CSH particles could be mixed with water at a liquid/powder ratio of 0.8 and the compressive strength of the product was 7.7. When the large particle‐size CSH are mixed with water at a ratio of 0.5, the gel‐like product is non‐injectable without compressive strength. Tang *et al*.^13^ studied the mixtures of water and CSH with different particles sizes and aspect ratios, and investigated the compressive strength. The aspect ratio, particle size, distribution value, and resulting compressive strength can be seen in Table 2. The data indicate that the compressive strength of particles with the identical aspect ratio and various sizes can result in similar compressive strength. When the aspect ratio ranges from 0.5 to 1 and 1 to 3, the compressive strength decreases as the ratio of particles sizing from 0 to 45 μm increases. When the aspect ratio ranges from 3 to 6, the compressive strength increases as the ratio of particles sizing from 0 to 45 μm increases.

This study also found that the compressive strength decreases as the diameter goes up. For example, the compressive strength of small‐particle CSH sample can reach 12 MPa, while that of a large‐particle sample can merely reach 6.6 MPa. But this is not to say that the sample with more small particles can obtain higher compressive strength, because its value might change according to a different ratio of the particle sizes. The maximum value obtained by this study is 14.16 MPa, much higher than the single‐sized samples, which resembles the study of Guan *et al*. The possible reason is that the small particles can be filled inside the voids of the large ones, leading to the CSD produce with a larger density and compressive strength. When CS‐based composites are prepared, the additive might affect mechanical properties of CS, and the variances of particle size distributions might further affect the formula and finally result in a solution to good compressive strength. Thus, this subject also provides theoretic supports to the study of CS‐based composites.

### *Effect of Particle Size on* In vivo *and* In vitro *Degradation Rate*

Roberts *et al*.^16^ studied the *in vitro* degradation rate of CS samples carrying antibiotics. The diameters of the samples studied are 4.8 mm and 3.0 mm respectively. When immersed into the same PBS solution, the dissolution rate of the particles with the diameter of 4.8 mm was relatively high compared to the 3.0 mm group (2.3 mg per day *vs* 1.3 mg per day). It took 30 days for the 3.0 mm particles to completely degrade, whereas 48 days were for the 4.8 mm particles. Chen *et al*.^17^ studied the effect of various modifiers to the particle sizes and degradation rate of CSH. The calculation simulated the *in vivo* composition of Ca^2+^ to analyze particle degradation. The research found that the group without modifier produced the largest size of particles, together with relatively low degradation rate. The group which applied sodium dodecyl benzene sulfonate as the modifier generated the smallest size of particles with the lowest degradation rate among all groups. The MgCl_2_ group produced relatively large particle sizes and the degradation is comparatively fast. The sodium citrate group obtained the lowest size of particles and highest degradation rate. These results suggested that, when single‐sized particles are used, a smaller size would induce higher degradation rate. However, it was also found that the absorption of the modifier would restrain the degradation process.

In addition, the particle size also affects the degradation rate *in vivo*. Zhang *et al*.^18^ implanted the nano‐ and micro‐scaled CS into the muscles at the left lower extremities of mice and observed its osteogenesis and degradation properties. The study found that nano‐scaled CS could promote the ingrowth of the blood vessel and the formation of new bone, and its absorption rate was obviously increased. In our study, it was observed that the CS with large particle size (75–150 μm) had the lowest absorption rate, while the middle and small particles were absorbed faster. In the mixtures with different particle size distributions, the *in vitro* degradation rate could be high at 50.7% when the weight percentages of small (0–37.5 μm), middle (37.5–75 μm), and large (75–150 μm) particles are 55.7%, 44.3%, and 0.00%, respectively. Thus, it can be concluded that 75 μm could be seen as a boundary of the particle size. The particle size over 75μm would induce slow absorption rate, while that below 75 μm could be absorbed relatively fast. This observation is mainly applied for the preparation of drug‐carrying CS, because the release speed of drug should be controlled within 1 month for a few patients with pyogenic osteomyelitis and spondylitis. The particle size distribution could be further adjusted to control the release speed.

### *Effect of Particle Size on Initial and Final Setting Time*

The particle size of CSH can affect its setting time. This is because a smaller particle size could induce large specific surface area and water‐contacting area, and this process accelerates the crystallization of water and shortens the setting time. This study found that both the initial and the final setting time prolong with the increase of particle size. The initial and final setting time of small‐particle samples are respectively 270 s and 380 s, while those of large‐particle ones are 410 s and 460 s, respectively. The reasonable distribution of particle sizes can reduce the setting time. It suggested that the minimum setting time was obtained when the percentages of 0–38 μm and 38–75 μm sized particles are 89.7% and 10.3%, respectively. This study provided the theoretic basis for CS‐based composites. This is because any types of additives would extend the setting time of CSH, further extending the operation time and inducing relative complication rate. Therefore, selecting an optimal ratio of CSH particle sizes is crucial to reduce setting time when the CS‐based composites are applied into research.

### *Effect of Particle Size on Osteogenesis*

The particle size of CS could also affect the osteogenetic abilities. Zhang *et al*.^18^ compared the osteogenesis between (i) nano‐grade CSD combined with DBM and (ii) micro‐grade CSD combined with DBM, and (iii) applied DBM only as the control group. Those three groups of materials are respectively implanted inside the muscles of mice, and HE staining method was used to observe the histochemical characteristics of the nude mice. After 28 days of the operation, all those three materials could be observed by the formation of bone, chondrocyte, ground substance, active osteoblast, etc. By the HE method, the new bone area found in the micro‐grade CSD was 16.0% ± 3.7%, while that in the nano‐grade CSD was 28.0% ± 4.6%. The control group resulted 12.0% ± 2.9%. Therefore, the nano‐grade CSD has better osteogenetic abilities and can be applied as an ideal bone graft material.

The related research also suggested that the surfaces of the nano‐structure was seen as the one mimicking natural tissues, such as glass adhesin and collagen. The nano‐grade particles can regulate the cellular proliferation of the skull osteoblasts and the expression of osteocalcin inside those cells. Besides, the substance with the nano‐scale morphology is thought to have essential signal‐conductive capability on the functional regulation of cellular migration, cellular polarization, and cytoskeletal. Hence the nano‐grade CSD was thought to possess favorable histo‐compatibility and osteoinductivity. Although this study did not further investigate the osteogenetic ability, it is believed that the favorable physical and chemical properties are the study basis of the biological properties. Only when the physical and chemical properties are assured to satisfy the study requirement can the biological properties, such as osteogenesis, be further studied.

### *Limitations of this Study*

1. This study did not study other biological trials, such as the cellular toxicity and intradermal simulation test. This is mainly because this subject is designed according to ASTM standards, and those aspects studied above are formal standards proposed for grafted surgical‐grade CS.
2. This study did not set up the control groups, such as the blank control or other samples with identical or similar applications. This is because the formula‐designing experiment is applied to set up several control groups among comparing groups, which can effectively confirm the relative preciseness of the experiment.
3. This subject selected “the degradation rate in 10 days” as the study targets and analyzed its effect by particle size ratios. The statistical analyses of other time periods are processed by other members of the research group, excluded from this subject.

## *Conclusion*

As for the initial and final setting time, a CSH sample with smaller size of particles has larger surface area, and is likely to have larger area to contact with water, and shorter reacting time with water, during the hydration process. On the contrary, a larger particle size of the CSH sample could lead to smaller surface area, smaller contacting area with water and longer time of reaction.

On the compressive strength, the smaller the particle size, the larger the surface area, and the larger the requirement of ionized water. In this term, there is little free water inside the sample, compared with a larger particle‐size CSH sample at the same solid–liquid ratio. After the sample is dried, the bulk sample could have smaller voids inside, resulting in higher compressive strength. If the large and small particles are used simultaneously, the small particles could be filled in the voids of the large particles at a proper particle ratio, and the void volume is further decreased, inducing the increase of the compressive strength.

On the degradation rate, a CS sample with larger particle sizes would result in smaller surface area and smaller contacting area with water. The dissolution rate of CSH into the surrounding is slowed down, causing slow degradation rate. On the contrary, a smaller particle size would result in larger contacting area with water, which is easier for CSH to be dissolved. This process would increase the degradation rate.

From the perspective of the long‐term research, although some other CS‐based composites have been confirmed to improve the characteristics of CS product, the original features of CS are still an essential condition to perform the physical and chemical properties. The morphology and structure of CS bulk material can affect the mechanical strength, resorption rate, while the particle size ratio is a significant aspect of the study. As this study only divided the particle sizes into three groups, further studies could examine a more detailed division of particle size groups in order to investigate more clearly its effect on the properties of CS materials.
